# Supplementary material for: Cerebral Organoids with Integrated Endothelial Networks Emulate the Neurovascular Unit and Mitigate Core Necrosis
Source: Adv Sci (Weinh). 2025 Aug 30;12(43):e07256. doi: 10.1002/advs.202507256 (PMC12631921; doi:10.1002/advs.202507256)
Supplement: Supplementary file 1 — Supporting Information [file ADVS-12-e07256-s001.docx]

**Supplementary Materials for**

**Cerebral Organoids with Integrated Endothelial Networks Emulate the Neurovascular Unit and Mitigate Core Necrosis**

Josep Fumadó Navarro^1,2^, Siobhan Crilly^1,2^, Wai Kit Chan^3^, Shane Browne^2,4,5^, John O. Mason^3^, Catalina Vallejo-Giraldo^2,6^, Abhay Pandit^2^, Mihai Lomora^1,2,7*^

^1^School of Biological and Chemical Sciences, College of Science and Engineering, University of Galway, University Road, Galway H91 TK33, Ireland

^2^CÚRAM, Research Ireland Centre for Medical Devices, University of Galway, Upper  Newcastle, Galway H91 W2TY, Ireland

^3^Simons Initiative for the Developing Brain, Centre for Discovery Brain Sciences, University of Edinburgh, Hugh Robson Building, George Square, Edinburgh EH8 9XD, UK

^4^Tissue Engineering Research Group, Department of Anatomy and Regenerative Medicine, Royal College of Surgeons in Ireland, 123, St Stephen’s Green, Dublin 2, Ireland

^5^Trinity Centre for Biomedical Engineering, Trinity College Dublin, Dublin 2, Ireland

^6^School of Engineering, College of Science and Engineering, University of Galway, University Road, Galway H91 TK33, Ireland

^7^Lead contact

*Correspondence: [mihai.lomora@universityofgalway.ie](mailto:mihai.lomora@universityofgalway.ie%22%20\\t%20%22_blank) (M.L.)

This section includes:

Figures S1-S10

Table S1-2


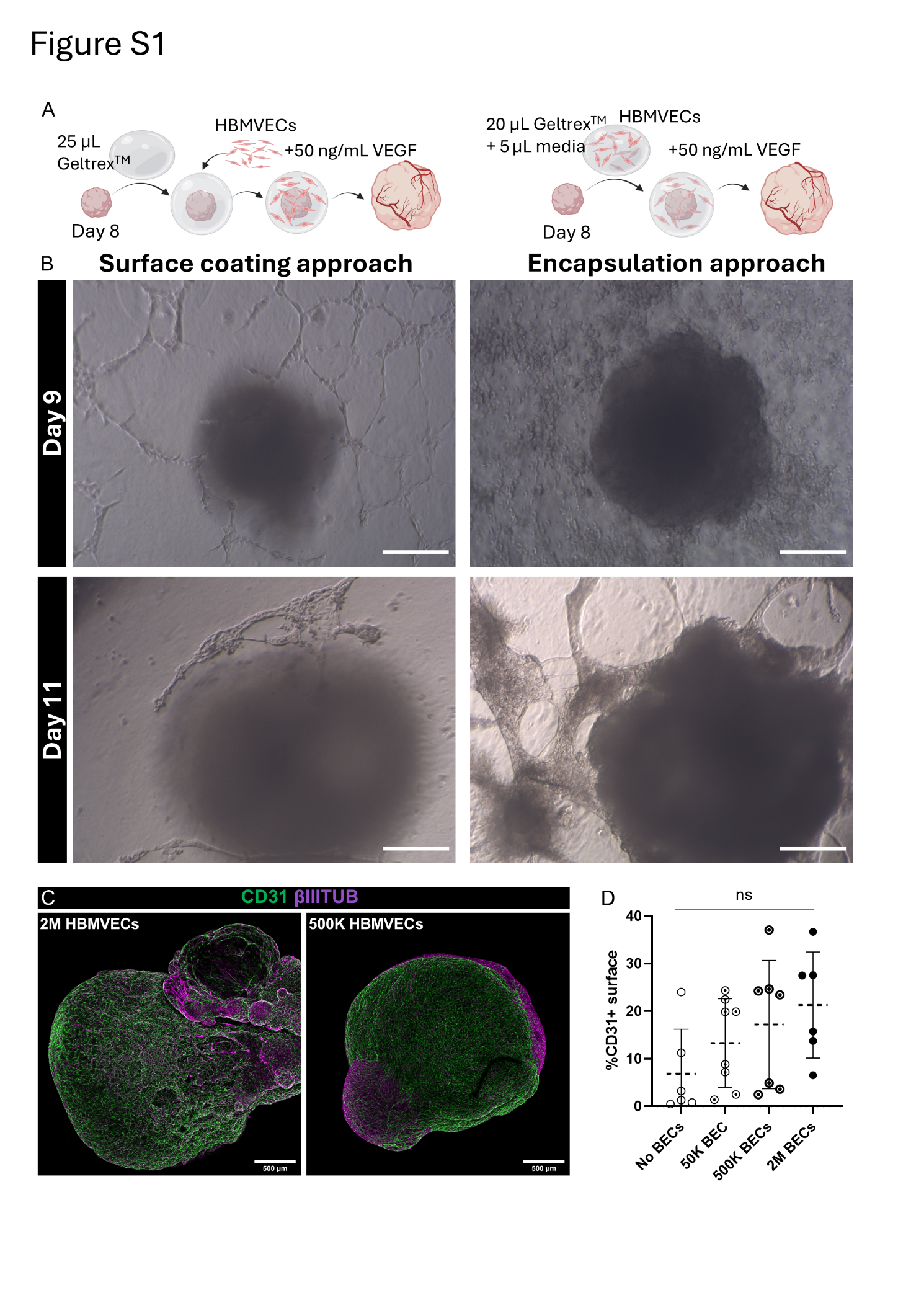


**Figure S1: Optimization of the vascularization strategy by testing two different HBMVECs incorporation methods and varying cell densities**

(A) Schematic representation of the surface coating approach consisting of adding HBMVECs after polymerizing the Geltrex^TM^ droplet embedding the organoid, allowing cell attachment (left panel). The encapsulation approach involved mixing HBMVECs with the biomaterial before embedding the organoid, followed by polymerization (right).

(B) Brightfield images showing 50,000 HBMVECs-organoid interactions in the first days after embedding (days 9 and 11) for both approaches. In the surface coating approach, endothelial networks are formed on the surface but deteriorate over time. In contrast, the encapsulation approach resulted in more stable network assemblies. Scale bars: 500 µm.

(C) Confocal images of A-COs grown with two different encapsulating cell densities (500,000 and 2 million HBMVECs), whole-stained for CD31 (green) and βIIITUB (magenta), depicting the organoid surface covered with HMBVECs. Scale bars: 500 µm.

(D) Percentage of CD31+ coverage on day 40 of A-COs grown with different encapsulating cell densities (0, 50,000, 500,000 and 2 million HBMVECs). Mean ± SD, N = 6-8 organoids, two independent batches, same A-iPSC line. One-way ANOVA test and Tukey’s post hoc comparisons; ns = no significant difference (p > 0.05).


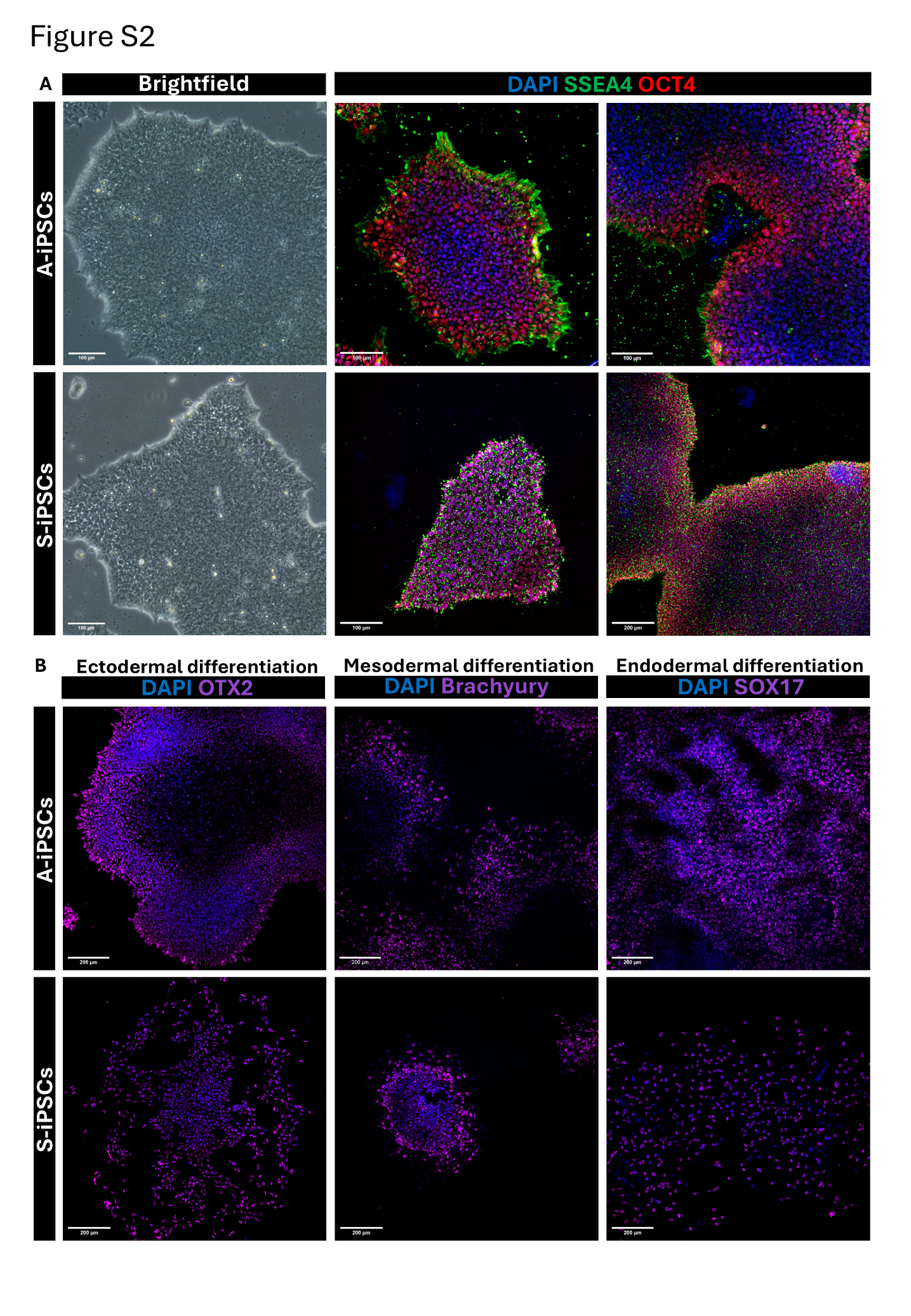


**Figure S2: Quality control assessment of two iPSC lines (A and S) through colony morphology evaluation, staining for pluripotent markers and a three germinal layer differentiation assay**

(A) Brightfield images of high-quality A- and S- iPSC cultures displaying compact colonies with well-defined edges. Confocal images of undifferentiated cultures stained for the pluripotency markers SSEA-4 (green), OCT4 (red), and nuclei (blue). Scale bars: 100 µm (bottom right image, 200 µm).

(B) Representative confocal images of ectodermal, mesodermal and endodermal progenitors (left to right) derived from A- and S-iPSC lines after a three-germinal-layer differentiation assay. Cells were immunostained for lineage-specific markers: OTX2 (ectoderm), brachyury (mesoderm), and SOX17 (endoderm). Scale bars: 200 µm


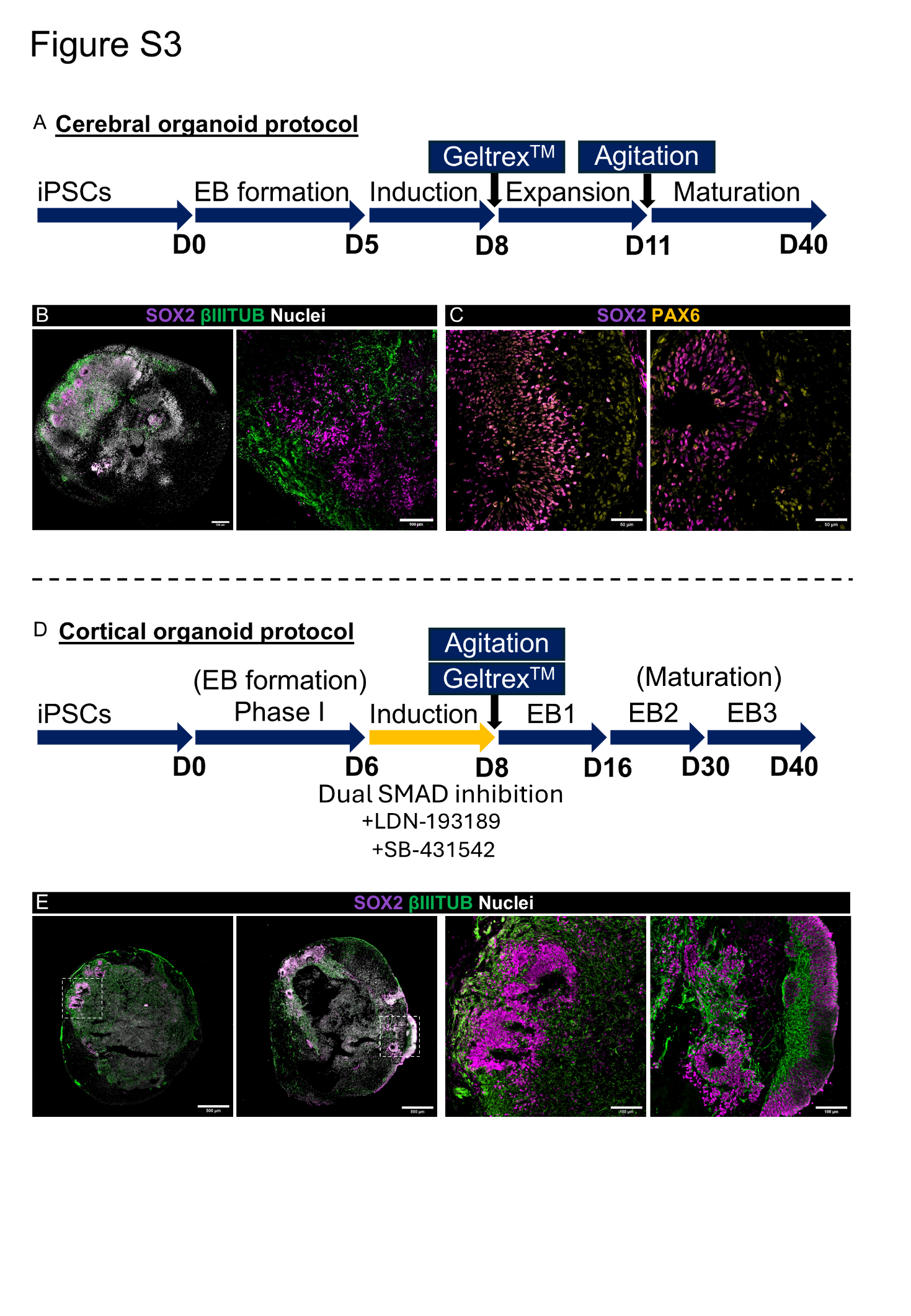


**Figure S3: Comparison of the cerebral organoid (CO) and cortical organoid (CortiCO) protocols used in this study, and examples of typical brain organoid features obtained with both methods**

(A) Schematic representation of key stages in the CO protocol.

(B) Confocal images of standard CO sections immunostained for SOX2 (neural stem cells, magenta), βIIITUB (neurons, green), and nuclei (white). Scale bars: 500 µm (left) and 100 µm (right).

(C) Confocal images of standard CO sections, showing neural rosettes immunostained for SOX2 (neural stem cells, magenta), PAX6 (radial glia, yellow), and nuclei (white). Scale bars: 50 µm.

(D) Schematic representation of key stages in the CortiCOs protocol.

(E) Confocal images of standard CortiCOs sections immunostained for SOX2 (neural stem cells, magenta), βIIITUB (neurons, green), and nuclei (white). Scale bars: 500 µm (left) and 100 µm (right).


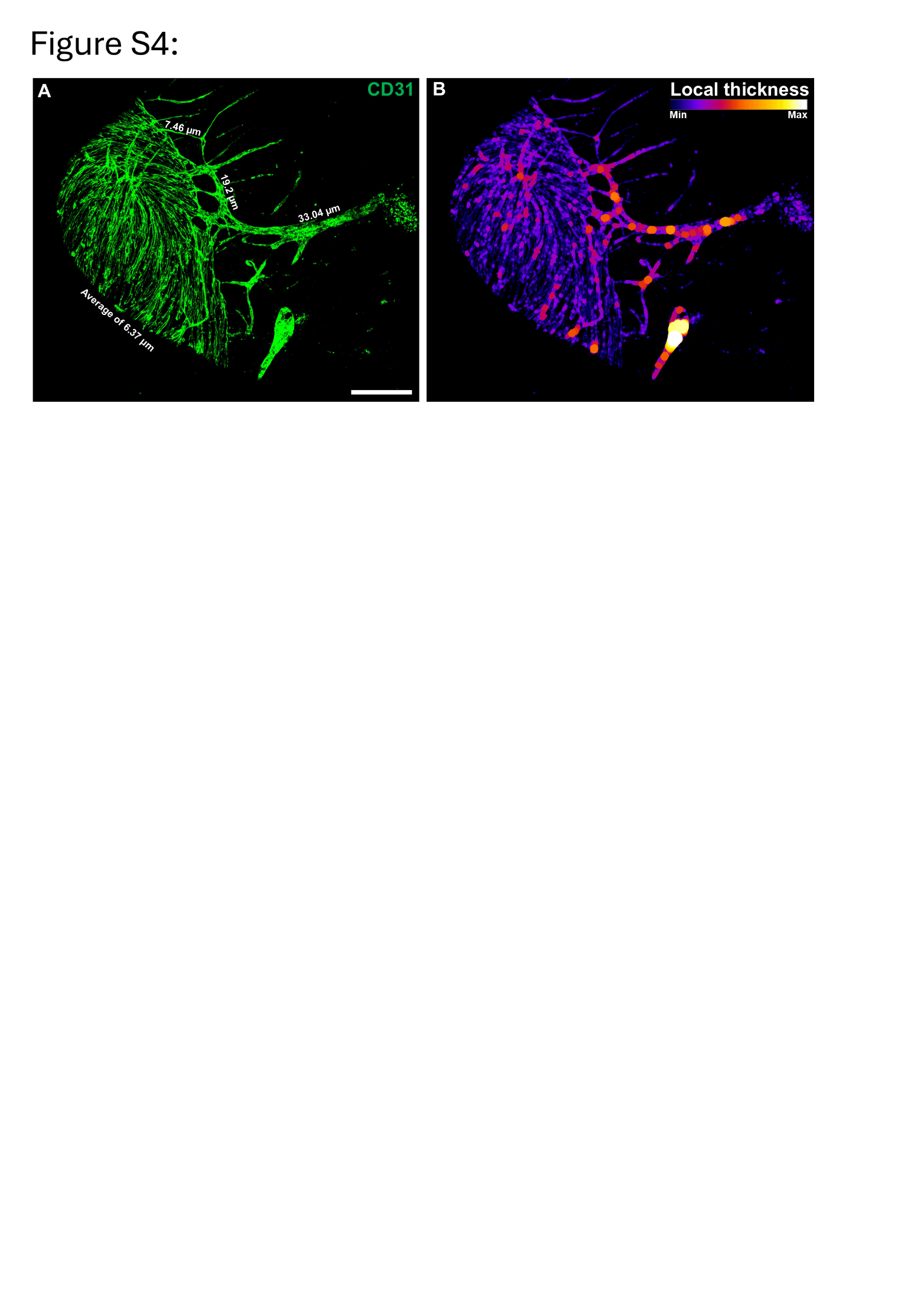


**Figure S4: Evidence of hierarchical morphology in some endothelial networks**

(A) Confocal image of a hierarchical endothelial network (CD31+) within a vascularized CO, showing a gradual decrease in vessel diameter from the right to the left. Scale bar: 200 µm.

(B) Visualization of vessel diameter variations using the Local Thickness plugin of ImageJ.


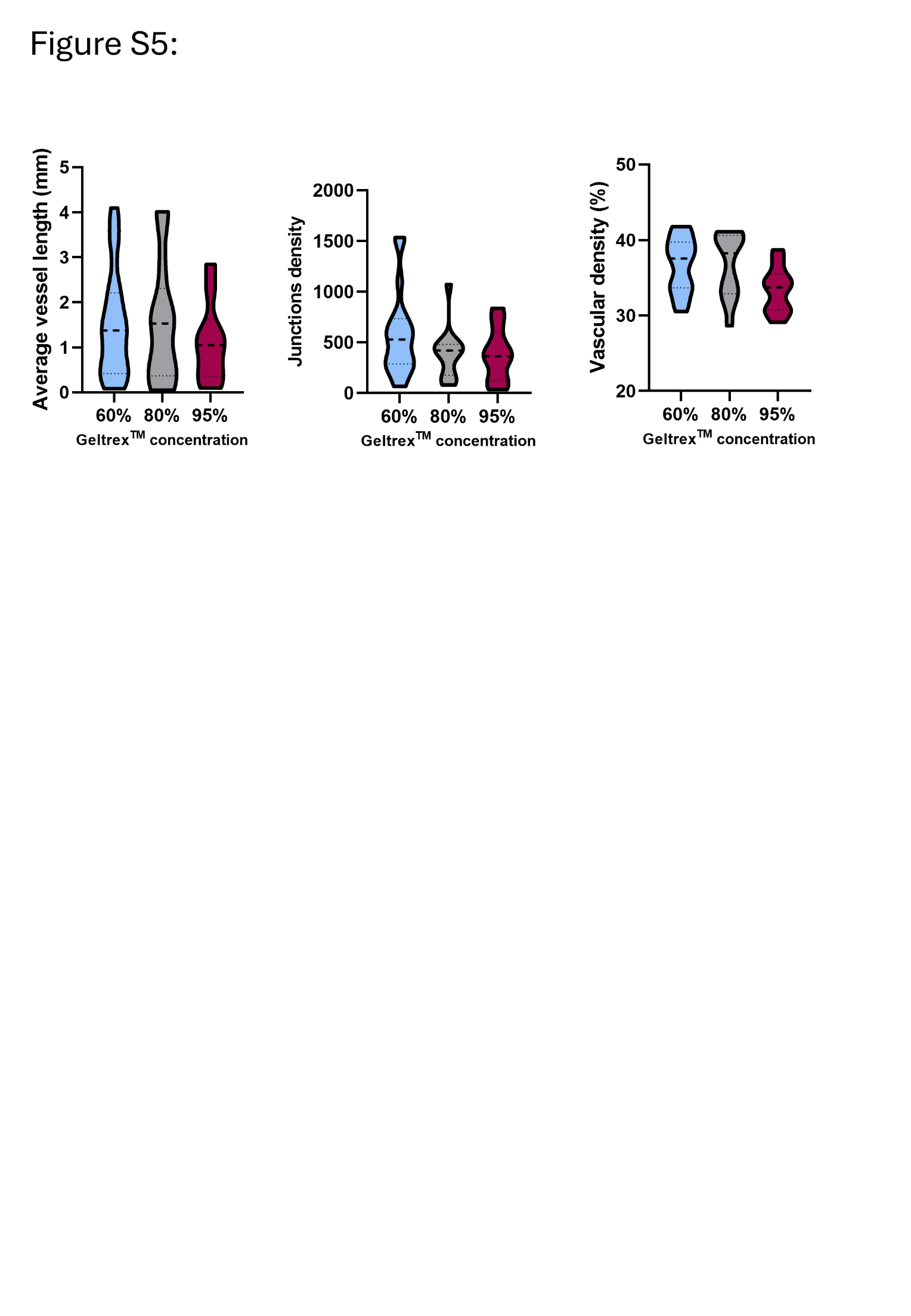


**Figure S5: Additional morphological analysis of superficial endothelial networks formed within different Geltrex^TM^ concentrations.** Quantification of junction density, average vessel length (mm), and vascular density (%) of superficial endothelial networks formed on organoids encapsulated with different Geltrex^TM^ concentrations. Light blue (60%), white (80%), Bordeaux (95%). Violin shape represents data distribution, Median (thick line), interquartile range (thin lines), N = 9-22, five different batches, same A-iPSC line. One-way ANOVA test and Tukey’s post hoc comparisons; not significant (p > 0.05).


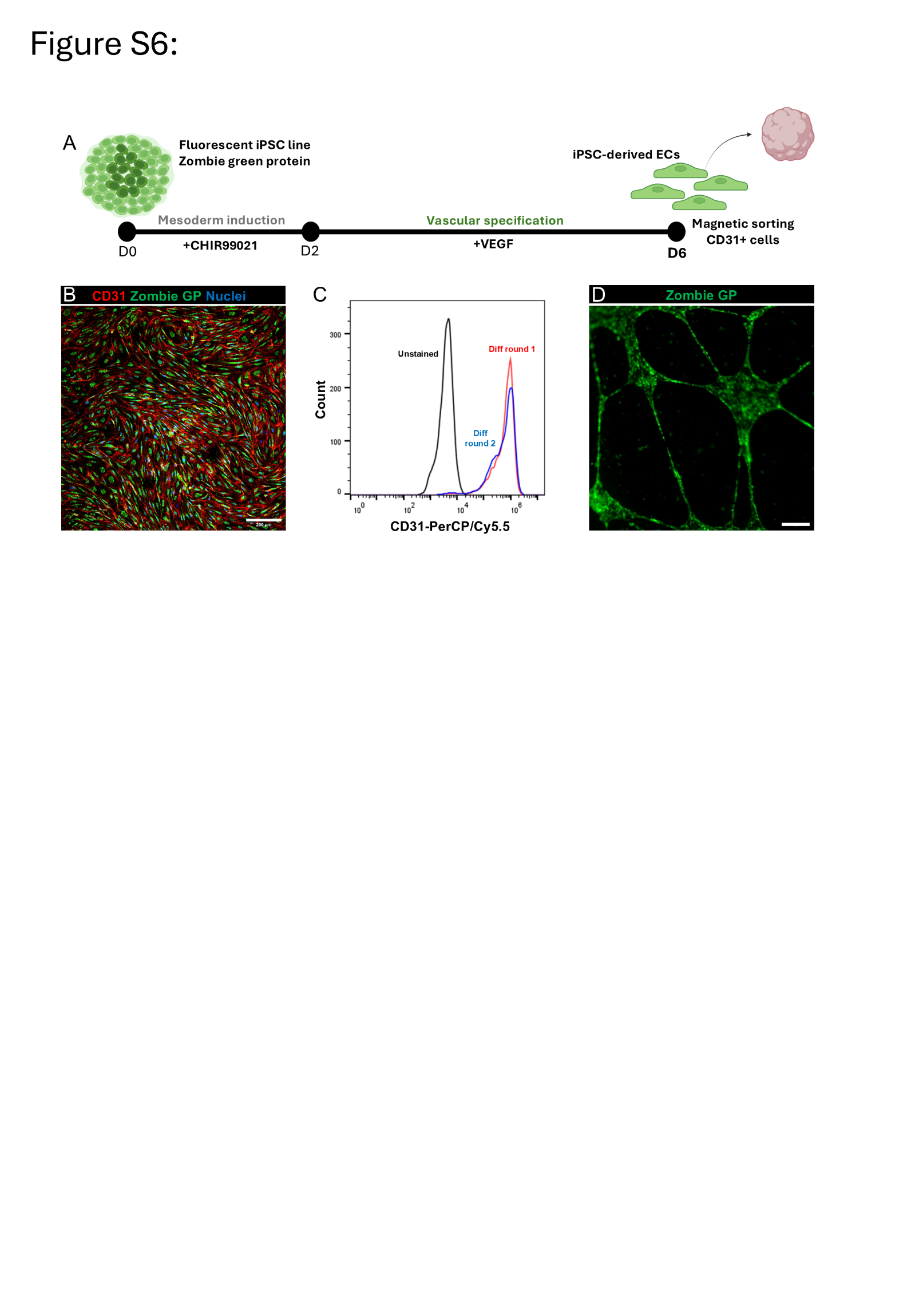


**Figure S6: Generation and characterization of fluorescent iPSC-derived endothelial cells (Z-ECs)**

(A) Schematic representation of the iPSC-to-endothelial cell differentiation protocol. Created with Biorender.com.

(B) Confocal image of fluorescent Z-ECs expressing Zombie Green protein in the cytoplasm, post-sorting. Cells were immunostained for CD31 (red) and nuclei (blue). Scale bar: 200 µm.

(C) Flow cytometry histogram of iPSC-derived ECs multiple passages after magnetic sorting. Black line, unstained cells; red and blue lines, two independent differentiations of cells labelled with a PerCP/Cy5.5-conjugated anti-CD31 antibody.

(D) Fluorescence microscopy image of a Z-ECs tubular assay on 100% Geltrex^TM^. Scale bar: 200 µm.


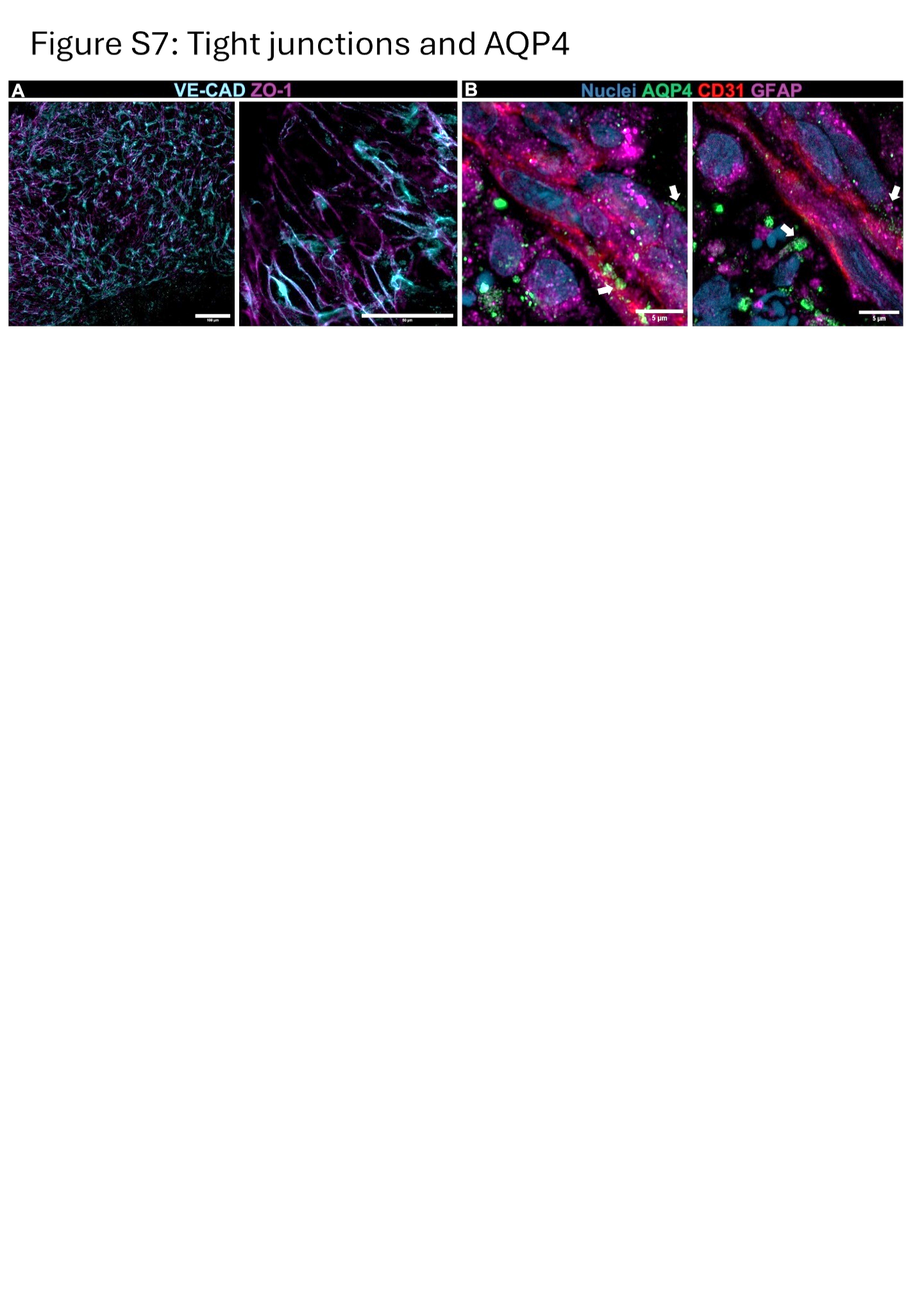


**Figure S7: Additional BBB characterization of the endothelial networks: immunostaining of the tight junction ZO-1 and the astrocytic end-foot marker Aquaporin 4 (AQP4).**

(A) Confocal images of the superficial endothelial networks of a vascularized A-CO whole-stained for VE-CAD (cyan) and ZO-1 (magenta). Scale bars: 100 μm (right) and 50 μm (left).

(B) Confocal images of a vascularized CO cryosection stained for AQP4 (astrocyte end-foot, green), GFAP (astrocyte, magenta), CD31 (endothelial tube, red) and nuclei (blue). White arrows point towards AQP4 staining in the proximity of an endothelial network. Scale bars: 5 μm.

**
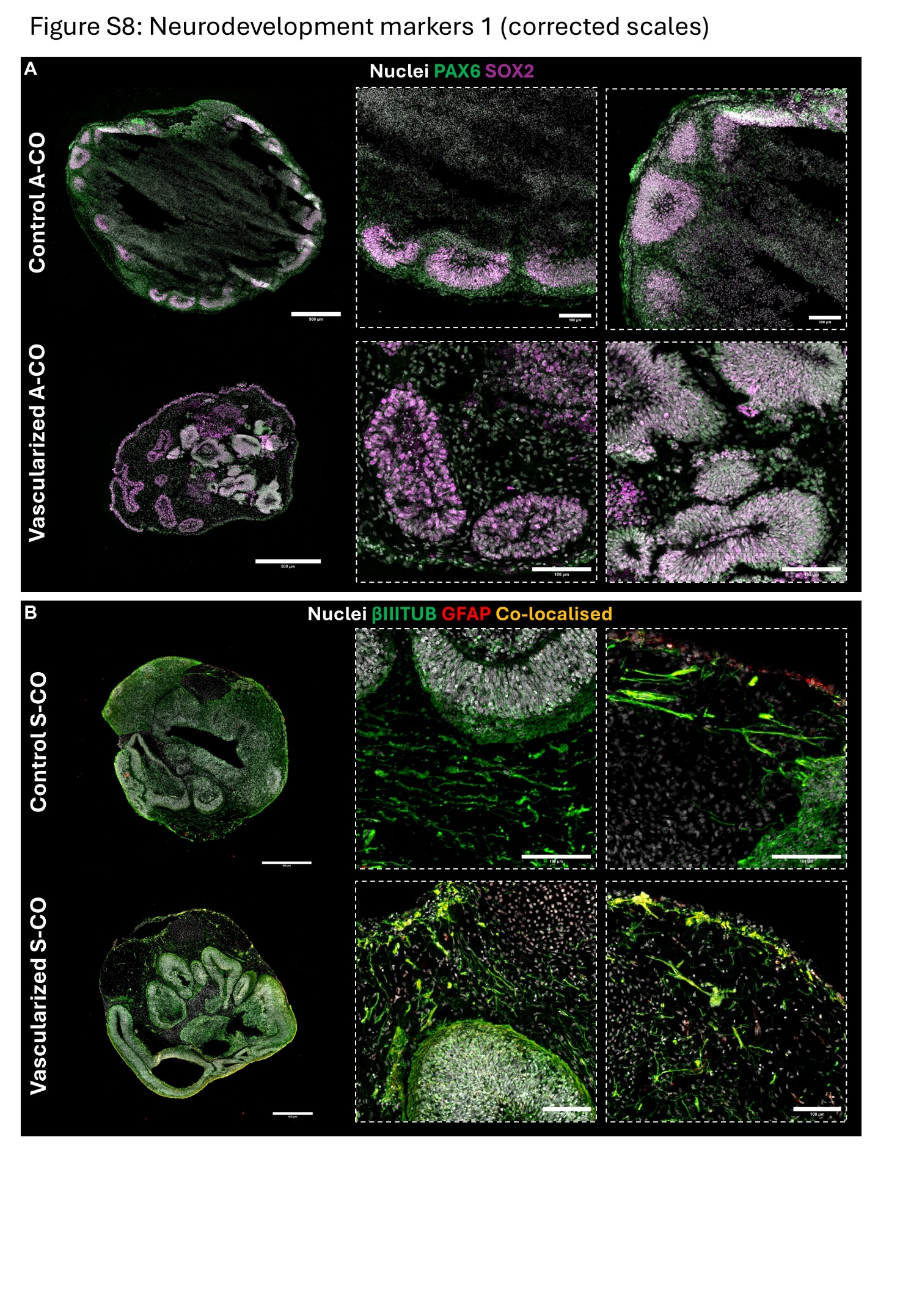
**

**Figure S8: Neurodevelopmental characterization of control and vascularized COs.**

(A) Representative confocal images of control (top) and vascularized (bottom) A-COs cryosections stained for SOX2 (neural stem cells, magenta), PAX6 (neuroprogenitor cells, green) and nuclei (white), depicting neural rosettes distribution and emergence of neuroprogenitor cells. Scale bars: 500 μm (left), 100 μm (middle and right).

(B) Representative confocal images of control (top) and vascularized (bottom) S-COs cryosections stained for βIIITub (neurons, green) and GFAP (astrocytes, red), revealing high abundance of neurons with similar axon distributions across conditions. Scale bars: 500 μm (left), 100 μm (middle and right).

**
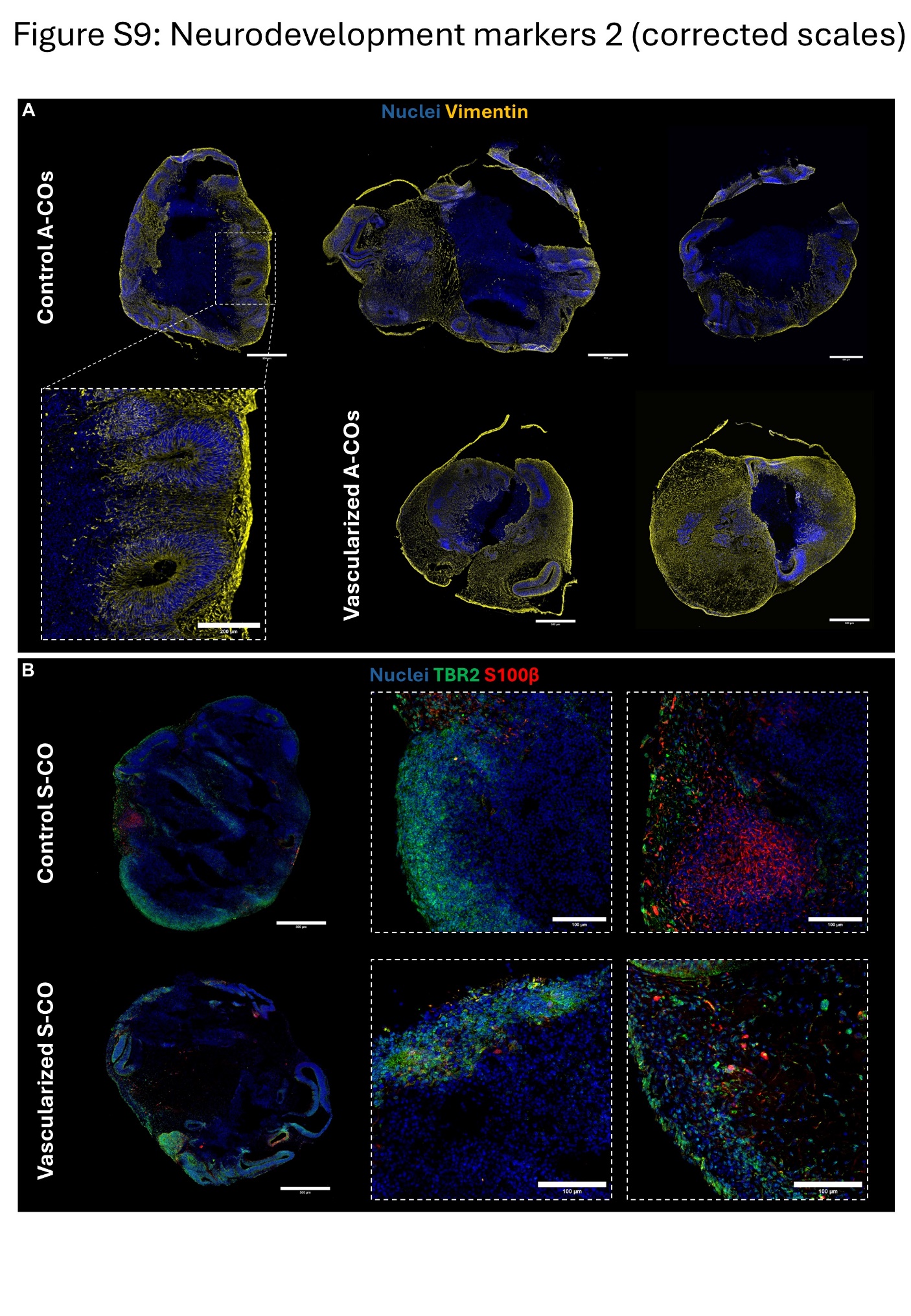
Figure S9: Additional neurodevelopmental characterization of control and vascularized COs.**

(A) Representative confocal images of control (top) and vascularized (bottom) A-COs cryosections stained for vimentin (radial glia, yellow) and nuclei (blue), showing radial glia coverage along the organoid and their absence in the necrotic core for both conditions. Scale bars: 500 μm and 200 μm (zoom in, bottom left).

(B) Representative confocal images of control (top) and vascularized (bottom) S-COs cryosections stained for TBR2 (intermediate progenitors, green) and S100β (early astrocytes, red), showing that TBR2+ layer is found, not abundantly, in both control and vascularized organoids, in addition to S100β cells clusters. Scale bars: 500 μm (left) and 100 μm (middle and right).

**
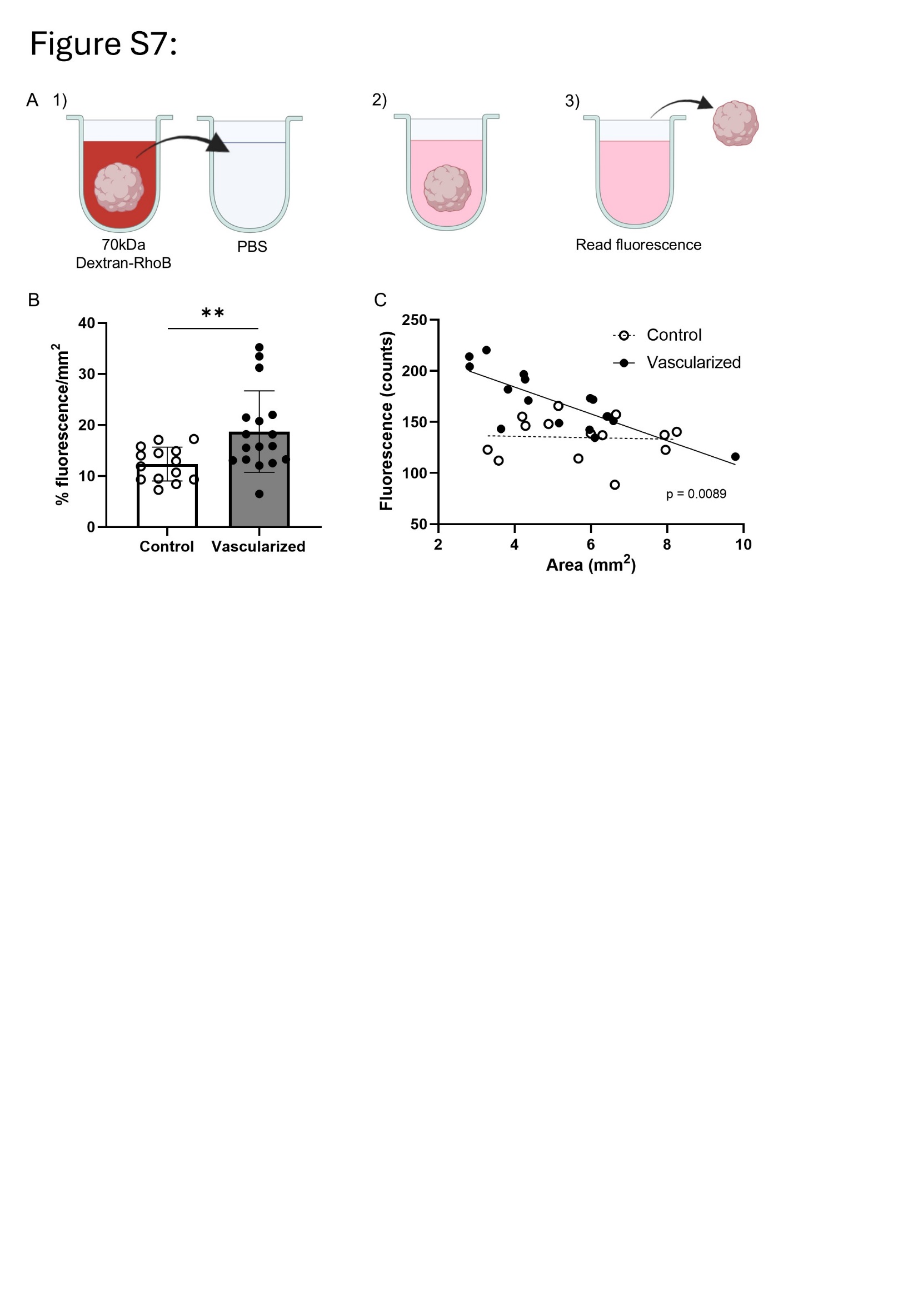
**

**Figure S10: 70 kDa dextran rhodamine B (d-RhoB) accumulation in control and vascularized COs and its correlation with organoid size**

(A) Schematic representation of the experimental workflow. 1) Organoids were incubated in d-RhoB solution and transferred with the same volume of media to a well filled with PBS. 2) Organoids were incubated with shaking to release the fluorescent content. 3) Organoids were removed with the same volume prior to fluorescence measurements ($\lambda_{ex}=570$, $\lambda_{ex}=595$ nm).

(B) Percentage of fluorescent counts transferred within the volume of d-RhoB containing the organoid, relative to the maximum fluorescence possible (same volume of d-RhoB solution alone), normalized by the area of the organoid (mm^2^). The same experiment was performed with control (white bar) and vascularized (grey bar) organoids from both A- and S-iPSC lines. Mean ± SD, N = 14-17, two independent batches, two iPSC lines. Welch’s T-test; **p < 0.01.

(C) Individual organoid sizes (mm^2^) and their respective fluorescence values. White dots (control organoids), black dots (vascularized organoids), and black line shows the linear regression trend. N = 14-15 per conditions, two independent batches, two iPSC lines. Regressions are significantly different: p = 0.0089.

100 μm

**Table S1. Information on reagents, cell lines, kits and software used in this study**

| **REAGENT or RESOURCE** | **SOURCE** | **IDENTIFIER** |
| --- | --- | --- |
| **Antibodies** |  |  |
| Mouse anti-human CD31 (PECAM-1) | Bio-Rad | Cat# MCA1738; RRID: AB_322710 |
| Rabbit anti-βIII-tubulin (βIIITUB) | Sigma-Aldrich | Cat# T3952; RRID: AB_1841226 |
| Mouse anti-βIII-tubulin (βIIITUB) | Sigma-Aldrich | Cat# T5293; RRID: AB_477580 |
| Rabbit anti-SOX2 | Invitrogen / Thermo Fisher Scientific | Cat# PA1-094; RRID: AB_2539862 |
| Mouse anti-PAX6 | Invitrogen / Thermo Fisher Scientific | Cat# MA1-109, RRID: AB_2536820 |
| Rabbit anti-human Collagen IV | Invitrogen / Thermo Fisher Scientific | Cat# PA5-95188, RRID: AB_2806993 |
| Mouse anti-human Laminin α5 | Invitrogen / Thermo Fisher Scientific | Cat# MA5-24649, RRID: AB_2637264 |
| Rabbit ant-Glial Fibrillary Acidic Protein (GFAP) | Agilent Dako | Cat# Z033401-2 |
| Goat anti-human brachyury (963427) | Bio-techne / R&D Systems | Included in the Human Pluripotent Stem Cell Functional Identification Kit; Cat# SC027B |
| Goat anti-human Otx2 (963273) |  |  |
| Goat anti-human SOX17 (963121) |  |  |
| Rabbit anti-VE-Cadherin | Cell Signaling Technology | Cat# 2500, RRID: AB_10839118 |
| Rabbit anti-PDGFRβ | Invitrogen / Thermo Fisher Scientific | Cat# MA5-15143, RRID: AB_10985851 |
| Mouse anti-α Smooth Muscle Actin (αSMA) | Abcam | Cat# ab7817, RRID: AB_262054 |
| Mouse anti-Human SSEA4 (A24867) | Thermo Fisher Scientific | Included in the Pluripotent Stem Cell Immunocytochemistry Kit (OCT4, SSEA4); Cat# A25526 |
| Rabbit anti-Human OCT4 (A24866) |  |  |
| Rabbit anti-aquaporin 4 (AQP4) | Novus biological | Cat# NBP1-87679 |
| Chicken anti-GFAP | Abcam | Cat# ab4674 |
| Rabbit anti-Vimentin | Abcam | Cat# ab92547 |
| Rabbit anti-EOMES (TBR2) | Invitrogen / Thermo Fisher Scientific | Cat# 15974054 |
| Mouse anti-S100 β-subunit | Sigma-Aldrich | Cat# S2532-100U |
| Mouse anti-ZO-1 | Invitrogen / Thermo Fisher Scientific | Cat# 339100 |
| Rabbit anti-VE-Cadherin | Abcam | Cat# ab33168 |
| Rabbit anti-Claudin 5 | Cell signaling | Cat# 49564 |
| Anti-human CD31 PerCP/Cyanine5.5 | BioLegend | Cat# 303132 |
| Donkey anti-mouse IgG (H+L) (Alexa Fluor™ 488) | Invitrogen / Thermo Fisher Scientific | Cat# A-21202, RRID: AB_141607 |
| Donkey anti-Rabbit IgG (H+L) (Alexa Fluor™ 594) | Invitrogen / Thermo Fisher Scientific | Cat# A-21207, RRID: AB_141637 |
| Goat anti-rabbit IgG (H+L) (Alexa Fluor™ 647) | Invitrogen / Thermo Fisher Scientific | Cat# A32733, RRID: AB_2633282 |
| Goat anti-mouse IgG (H+L) (Alexa Fluor™ 647) | Invitrogen / Thermo Fisher Scientific | Cat# A-21236, RRID: AB_2535805 |
| Donkey anti-goat IgG H&L (Alexa Fluor™ 647) | Abcam | Cat# ab150131; RRID: AB_2732857 |
| Goat anti-chicken IgG H&L (Alexa Fluor™ 647) | Invitrogen / Thermo Fisher Scientific | Cat# A21449 |
| **Chemicals, peptides, and recombinant proteins** |  |  |
| Essential 8™ Flex Medium Kit | Thermo Fisher Scientific | Cat# A2858501 |
| Geltrex™ Reduced-Growth Factor Basement-Membrane Matrix, LDEV-free, stem-cell qualified | Thermo Fisher Scientific | Cat# A1413302 |
| RevitaCell™ Supplement (100X) | Gibco / Thermo Fisher Scientific | Cat# A2644501 |
| Gentle Cell Dissociation Reagent | STEMCELL technologies | Cat# 100-0485 |
| KnockOut™ DMEM | Gibco / Thermo Fisher Scientific | Cat# 10829018 |
| Endothelial Cell Growth Medium | PromoCell | Cat# C-22110 |
| Gelatin, from porcine skin | Sigma-Aldrich | Cat# G2500-500G |
| Trypsin-EDTA Solution | Sigma-Aldrich | Cat# T4049 |
| Dimethyl sulfoxide (DMSO) | Sigma-Aldrich | Cat# D2650-100ML |
| Human Recombinant VEGF-165 | STEMCELL technologies | Cat# 78073 |
| Calcein AM | Invitrogen / Thermo Fisher Scientific | Cat# C3100MP |
| OCT compound | VWR Chemicals | Cat# 361603E |
| Fluoromount™ Aqueous Mounting Medium | Sigma-Aldrich | Cat# F4680-25ML |
| D-Sucrose | Thermo Fisher Scientific | Cat# BP220-1 |
| Hanks’ Balanced Saline Solution | Sigma-Aldrich | Cat# H9269-500ML |
| Dulbecco′s Phosphate Buffered Saline | Sigma-Aldrich | Cat# D8537-500ML |
| DMEM/F-12, HEPES | Gibco / Thermo Fisher Scientific | Cat# 11574546 |
| KnockOut™ Serum replacement | Gibco / Thermo Fisher Scientific | Cat# 10829220 |
| Fetal Bovine Serum | Sigma-Aldrich | Cat# F7524 |
| GlutaMAX™ Supplement | Gibco / Thermo Fisher Scientific | Cat# 35050061 |
| MEM Non-Essential Amino Acids Solution (100X) | Gibco / Thermo Fisher Scientific | Cat# 11140050 |
| Penicillin-Streptomycin | Sigma-Aldrich | Cat# P0781 |
| 2-Mercaptoethanol | Sigma-Aldrich | Cat# M3148 |
| Recombinant Human FGF2 (145 aa) Protein | R&D Systems | Cat# 3718-FB |
| Y-27632 (Dihydrochloride) | STEMCELL Technologies | Cat# 72302 |
| B-27™ Supplement (50X), minus vitamin A | Gibco / Thermo Fisher Scientific | Cat# 12587010 |
| Heparin sodium salt from porcine intestinal mucosa | Sigma-Aldrich | Cat# H3149-10KU |
| SB 431542 hydrate | Sigma-Aldrich | Cat# S4317-5MG |
| LDN193189 hydrochloride | Sigma-Aldrich | Cat# SML0559-5MG |
| Advanced DMEM/F12 | Gibco / Thermo Fisher Scientific | Cat# 11540446 |
| N-2 supplement | Thermo Fisher Scientific | Cat# 11520536 |
| Neurobasal™ Medium | Gibco / Thermo Fisher Scientific | Cat# 11570556 |
| B27 Supplement 50X serum free | Thermo Fisher Scientific | Cat# 11530536 |
| Insulin solution human | Sigma-Aldrich | Cat# I9278-5ML |
| Recombinant human NT3 | PeproTech / Thermo Fisher Scientific | Cat# 450-03 |
| Human/Mouse/Rat BDNF Recombinant Protein | PeproTech / Thermo Fisher Scientific | Cat# 450-02 |
| Anti-Adherence Rinsing Solution | STEMCELL Technologies | Cat# 07010 |
| Advanced DMEM | Thermo Fisher Scientific | Cat# 12491015 |
| L-Ascorbic Acid | Sigma-Aldrich | Cat# A5960-25G |
| Laduviglusib (CHIR-99021) | MedChemExpress / Thermo Fisher Scientific | Cat# 16041282 |
| Invitrogen™ Dynabeads™ Sheep Anti-Mouse IgG | Invitrogen / Thermo Fisher Scientific | Cat# 10630514 |
| Rhodamine B isothiocyanate–Dextran | Sigma-Aldrich | Cat# R9379-250MG |
| Agarose, low gelling temperature | Sigma-Aldrich | Cat# A9414-10G |
| Hoechst 33342 solution, 20 mM | Thermo Fisher Scientific | Cat# 62249 |
| 7-Aminoactinomycin D (7-AAD) | Thermo Fisher Scientific | Cat# A1310 |
| Paraformaldehyde | Sigma-Aldrich | Cat# 158127-500G |
| Ethanol | Honeywell | Cat# 32221 |
| Bovine serum albumin | Sigma-Aldrich | Cat# A2153-50G |
| Triton^TM^ X-100 | Sigma-Aldrich | Cat# T878-250ML |
| Trisodium citrate dihydrate | Sigma-Aldrich | Cat# S1804-500G |
| Tween^TM^ 20 | MP biomedicals | Cat# TWEEN201 |
| NaCl | Sigma-Aldrich | Cat# S9888 |
| MgCl_2_ | Sigma-Aldrich | Cat# M8266 |
| HEPES | Sigma-Aldrich | Cat# H4034 |
| Xylenes 98% | Thermo Fisher Scientific | Cat# 383930050 |
| DPX mountant for histology | Sigma-Aldrich | Cat# 06522-500ML |
| Hematoxylin solution (Mayer’s hemalum solution) | Sigma-Aldrich | Cat# 1.09249-500ML |
| Eosin Y solution | Thermo Fisher Scientific | Cat#  10562614 |
| Tetrahydrofuran | Sigma-Aldrich | Cat# 360589-500ML |
| Triethylamine | Sigma-Aldrich | Cat# 471283 |
| Dibenzyl ether | Thermo Fisher Scientific | Cat# 10113630 |
| **Critical commercial assays** |  |  |
| STEMdiff™ Cerebral Organoid Kit | STEMCELL Technologies | Cat# 08570 |
| Pluripotent Stem Cell Immunocytochemistry Kit (OCT4, SSEA4) | Thermo Fisher Scientific | Cat# A25526 |
| Included in the Human Pluripotent Stem Cell Functional Identification Kit | Bio-techne / R&D Systems | Cat# SC027B |
| Human MMP-9 ELISA Kit | Proteintech | Cat# KE00164 |
| Human VEGF ELISA Kit - Quantikine | Bio-techne / R&D Systems | Cat# SVE00 |
| Trichrome Stain (Masson) Kit | Sigma-Aldrich | Cat# HT15 |
| Click-iT™ Plus TUNEL Assay Kits for In Situ Apoptosis Detection | Invitrogen / Thermo Fisher Scientific | Cat# 15350244 |
| MycoStrip® Mycoplasma Detection Kit | InvivoGen | Cat# rep-mysnc-50 |
| **Experimental models: Cell lines** |  |  |
| Gibco™ Human Episomal iPSC Line (A-iPSCs) | Gibco / Thermo Fisher Scientific | Cat# A18945 |
| Healthy Control Human iPSC Line SCTi003-A (S-iPSCs) | STEMCELL technologies | Cat# 200-0511 |
| Human brain microvascular endothelial cells | Innoprot | Cat# P10361 |
| **Software and algorithms** |  |  |
| GraphPad Prism 8.0.2 | GraphPad | https://www.graphpad.com/scientificsoftware/prism/ |
| Angiotool | Public (NIH) | https://ccrod.cancer.gov/confluence/display/ROB2/Downloads |
| ImageJ 1.54f | Public (NIH) | https://imagej.net/ij/ |
| Optics11 DataViewer V2.5.0 | Optics11 life | https://www.optics11life.com/products/pavone-nanoindenter/#applications |
| QuPath-0.4.3 | Public (GNU) | https://qupath.github.io/ |
| Aperio ImageScope | Leica Biosystems | https://www.leicabiosystems.com/digital-pathology/manage/aperio-imagescope/ |
| Fluoview software FV31S | Evident | https://evidentscientific.com/en/software/cellsens |
| Fusion software | OXFORD instruments | https://fusion-benchtop-software-guide.scrollhelp.site/fusionum/ |
| FlowJo v10 | BD Biosciences | https://www.flowjo.com/flowjo/download |

**Table S2. Solutions and media compositions**

| Name | Composition |
| --- | --- |
| Permeabilization buffer | 300 µM sucrose, 50 µM NaCl, 6.3 µM MgCl_2_, 20 µM HEPES, 0.5% Triton X-100, pH 7.2 in distilled water |
| Phase I media | DMEM F-12 with HEPES, 20% KOSR, 3% FBS, 1% Glutamax, 1% MEM-NEAA, 1% Pen/Strep, 0.1 mM 2-mercaptoethanol, 4 ng/mL fibroblast growth factor 2 (FGF2) and 50 µM ROCK inhibitor |
| Neural induction media | DMEM F-12 with HEPES, 20% KOSR, 1% Glutamax, 1% MEM-NEAA, 1% Pen/Strep, 0.1 mM 2-mercaptoethanol, 10 µM SB431542 and 100 nM LDN-193189 |
| EB1 medium | Advanced DMEM/F12, 1% N2, 1% Glutamax, 1% Pen/Strep, 0.5% B27 without vitamin A, 4 µg/mL Heparin |
| EB2 media | 50% Advanced DMEM/F12, 50% Neurobasal media, 1% N2, 1% Pen/Strep, 1% MEM-NEAA, 0.5% Glutamax, 0.5% B27 without vitamin A, 2.5 µg/mL insulin) |
| EB3 media | 50% Advanced DMEM/F12, 50% Neurobasal, 0.5% N2, 1% Pen/Strep, 0.5% MEM-NEAA, 1% Glutamax, 1% B27, 2-mercaptoethanol, 5 µg/mL insulin, 20 ng/mL NT3 and 20 ng/mL BDNF |
| LARS media | Advanced DMEM, 60 µg/mL ascorbic acid, 2.5 mM Glutamax |
| Sodium citrate buffer | 10 mM sodium citrate, 0.05% Tween 20, pH 6, in PBS |
| FACS buffer | 2% FBS in PBS |
